# "Official" or "Unofficial"

**Published:** 1898-02-26

**Authors:** 


### EDITOR'S LETTER-BOX.

[Our Correspondents are reminded that prolixity is a great bar to publication, and that brevity of style and conciseness of statement greatly facilitate early insertion.]

### "OFFICIAL" OR "UNOFFICIAL."

SIR,—In an editorial article with the above heading in THE HOSPITAL of February 5th, the President of Council of the British Medical Association is criticised for the defence he made at the recent meeting of Council in reply to the accusation of Drs. Woodcock and Beverley. As this article seems to me to be in parts unfair towards Dr. Saundby, and as he can scarcely answer it, I ask space for a few comments thereon.

The line pursued in that article is that the President would have been completely justified in "officially" assisting the candidature of Sir Walter Foster; and, in spite of Dr. Saundby's own assertion to the contrary, the writer still considers that his action throughout the contest was carried on in his official capacity of President of Council. So far the article approves his conduct. But it afterwards complains because, in reply to his accusers, "Dr. Saundby was apologetic," and "instead of maintaining his right, and in fact, his duty to act for the Association in an emergency, denied that soft impeachment, and stated that he 'did not at any time entertain the thought of interfering officially in that election.'" I submit, that you take advantage of a little verbal slip when you speak of the President's "waiting" as an "official" action. There is, of course, an ellipsis; the quotation "felt that officially there was nothing to be done but to wait" should run that he "felt that officially there was nothing to be done, and that he could only await events." Lower down you remark—somewhat unfairly, I consider, because you thus beg the whole question—that "the President of Council then addressed letters to the secretaries of branches," &c. Further on, in arguing that Dr. Saundby's action was "official," the article says, "that the acts of the President of the Council, the elect of the elected, the executive head of the Association must be official when they have to do with Association affairs" Quite right; but the election to the General Medical Council does not come under that heading of "Association affairs." Lastly, as to the assertion that Dr. Saundby should have boldly justified his action as "official," he could only have done so, it seems to me, by being false to his own opinions. The position taken up by Dr. Saundby in his defence was not cowardly, but was the only position consistent with truthfulness.—I am, &c., MEMBER OF COUNCIL (B.M.A).

February 10th, 1898.

*** The writer of the above letter is entirely in error. So far from it being the case that we considered Dr. Saundby's action to have been official, the very point of our protest was that we considered that the admission by the Council that such a wide latitude of unofficial action was permissible created "a very awkward precedent." "Member of Council" says that we took advantage of a little verbal slip. We took no advantage of anything or anybody, but followed, word for word, the official report given in the *British Medical Journal,* published more than a week after the meeting, time enough surely to have obtained an accurate record of what had passed. Why it is "unfair" for us to say that "the President of the Council then addressed letters to the secretaries of branches" we do not know. It was not, as "Member of Council" calls it, "begging the question"; the thing was admitted, and was the very point that had given rise to the discussion. We did not blame Dr. Saundby for writing these letters; all we have said is that when things had been done which, to the protesting members of Council, looked so very like official acts, the acceptance by the Council of the explanation that they were done unofficially "created a very serious precedent as to the liberty of official personages to perform unofficial acts pertaining to their office" during their year of service. We certainly cannot congratulate Dr. Saundby on his advocate. His words suggest that some people might think Dr. Saundby's action "cowardly," a thing we never suggested or thought of suggesting in the slightest degree; and as to his statement that the election to the General Medical Council does not come under the "heading of 'Association affairs,'" we can but say that the case of Dr. Saundby must, in the opinion of "Member of Council," be very far gone indeed if he thinks the acceptance of such a proposition is necessary for its defence. What, indeed, are we to think of the virility of the British Medical Association when a member of its Council can maintain in defence of the action of the President of its Council that intervention in the election of a direct representative on the General Medical Council is not an Association affair?—ED. *T. H.*

